# Achilles tendon ruptures during summer show the lowest incidence, but exhibit an increased risk of re-rupture

**DOI:** 10.1007/s00167-020-05982-x

**Published:** 2020-04-20

**Authors:** I. A. Saarensilta, G. Edman, P. W. Ackermann

**Affiliations:** 1 Department of Molecular Medicine and Surgery, Karolinska Institutet, Stockholm, Sweden; 2 Department of Psychiatry, Tiohundra AB, Norrtälje, Sweden; 3 Department of Orthopedic Surgery, Karolinska University Hospital, 171 76 Stockholm, Sweden

**Keywords:** Epidemiology, Seasons, Deep venous thrombosis, Infection, Seasonal variation, Achilles tendon Total Rupture Score

## Abstract

**Purpose:**

Achilles tendon rupture (ATR) is a common injury. The knowledge of seasonal factors´ impact is incomplete, but may provide means for preventive approaches for Achilles tendon related morbidity. The aim of this study was to investigate seasonal variations in ATR incidence in relation to injury mechanism, adverse events including risk of re-rupture, and patient-reported outcome in adults in Stockholm, Sweden.

**Methods:**

In total, 349 patients with unilateral acute Achilles tendon rupture, prospectively treated with standardized surgical techniques, were retrospectively assessed. Date of injury was assigned to one of the four internationally defined meteorological seasons in the northern hemisphere. Injury mechanism and the rate of adverse events; deep venous thrombosis, infection and re-rupture in relation to per-operative complications. Patient-reported outcome at 1 year was assessed with the validated Achilles tendon Total Rupture Score.

**Results:**

ATR incidence was significantly highest during winter and spring, and lowest during summer (*p* < 0.05). The most common sporting activities associated with ATR were badminton, floorball and soccer (> 50%). The rate of soccer-related ATR was highest during summer (*p* < 0.05). Patients sustaining an ATR during summer, compared to other seasons, exhibited more per-operative complications (*p* < 0.05), a significantly higher risk of re-rupture (*p* < 0.05) and a lower rate of good outcome (n.s.). The risk of other adverse events after ATR did not differ between the seasons.

**Conclusion:**

Winter and spring are the high risk seasons for sports-related ATR and the risk sports are badminton, soccer and floorball. The reason for the higher risk of re-rupture after ATR repair during summer should be further investigated.

**Level of evidence:**

III.

## Introduction

Achilles tendon rupture (ATR) is increasing in incidence, which emphasizing the need to improve the understanding of associated risk factors to inform preventive strategies, such as patient education [9, 14]. Additionally, patient outcome after ATR healing is still suboptimal and heterogenous and the risk of adverse events is high. Seasonal factors may impact both the risk of ATR and its adverse events, but the knowledge is limited. Thus, increased knowledge on seasonal patterns is warranted and may help in the development of new clinical practices for decreasing ATR-related morbidity.

In register-based studies from Vancouver and New York City, sports-related ATR was most common during spring, but there was no seasonal relationship to non-sports-related ATR [3, 24]. In contrast, a large nationwide register-based Danish study showed highest incidence of ATR during autumn [9]. Thus, differences between countries seem to exist and there is a lack of Swedish studies within this topic. Whether a season-related increased risk of ATR could be identified and related to a specific sport, this would provide the means to start sports-specific prevention during or even before the high-risk season.

Acute ATR is most commonly related to sports [15, 24, 25], and especially to sporting activities involving explosive movements of the calf muscles [11]. The specific sporting activities causing ATRs seem to vary between countries [15]. The most common causes for ATR have been badminton and soccer in Denmark, volleyball in Finland [12, 21], basketball and tennis in USA [22], and soccer and volleyball in Canada [25]. No earlier Swedish studies on sporting activities associated with ATR exist.

Deep venous thrombosis (DVT) is a major complication, which affects up to 50% of ATR-patients, treated operatively or conservatively [6, 7, 10, 17, 20, 23]. Suffering a DVT has been demonstrated to result in impaired patient-reported outcome at 1 year post ATR [5]. A large meta-analysis showed an increased incidence of venous thromboembolism during winter, without specification of the underlying cause [4]. Whether seasonal factors could affect discrepancies in patient outcome as well as DVT-rate after ATR has been unknown.

Furthermore, infections and re-ruptures may also be associated with seasonal factors and affect the patient outcome. It has been suggested that the rate of surgical site infections in orthopaedic foot and ankle surgery is higher during summer, but differences were not statistically significant [13]. Whether seasonal variation in the risk of re-rupture after an ATR reconstruction exists or not, has been unknown.

A retrospective cohort study was conducted with the aim to investigate seasonal variations in the rate of ATR in relation to injury mechanisms, risk of DVT, risk of infection, risk of re-rupture in relation to treatment factors, and seasonal variation in patient-reported outcome in adults in Stockholm, Sweden.

## Materials and methods

This study was conducted with an approval from the Regional Ethical Review Committee in Stockholm, Sweden (ID nr. 2009/2079-31/2, 2013/1791-31/3). All participants received oral and written information about the study procedure and provided written informed consent prior to surgery.

**Fig. 1:**
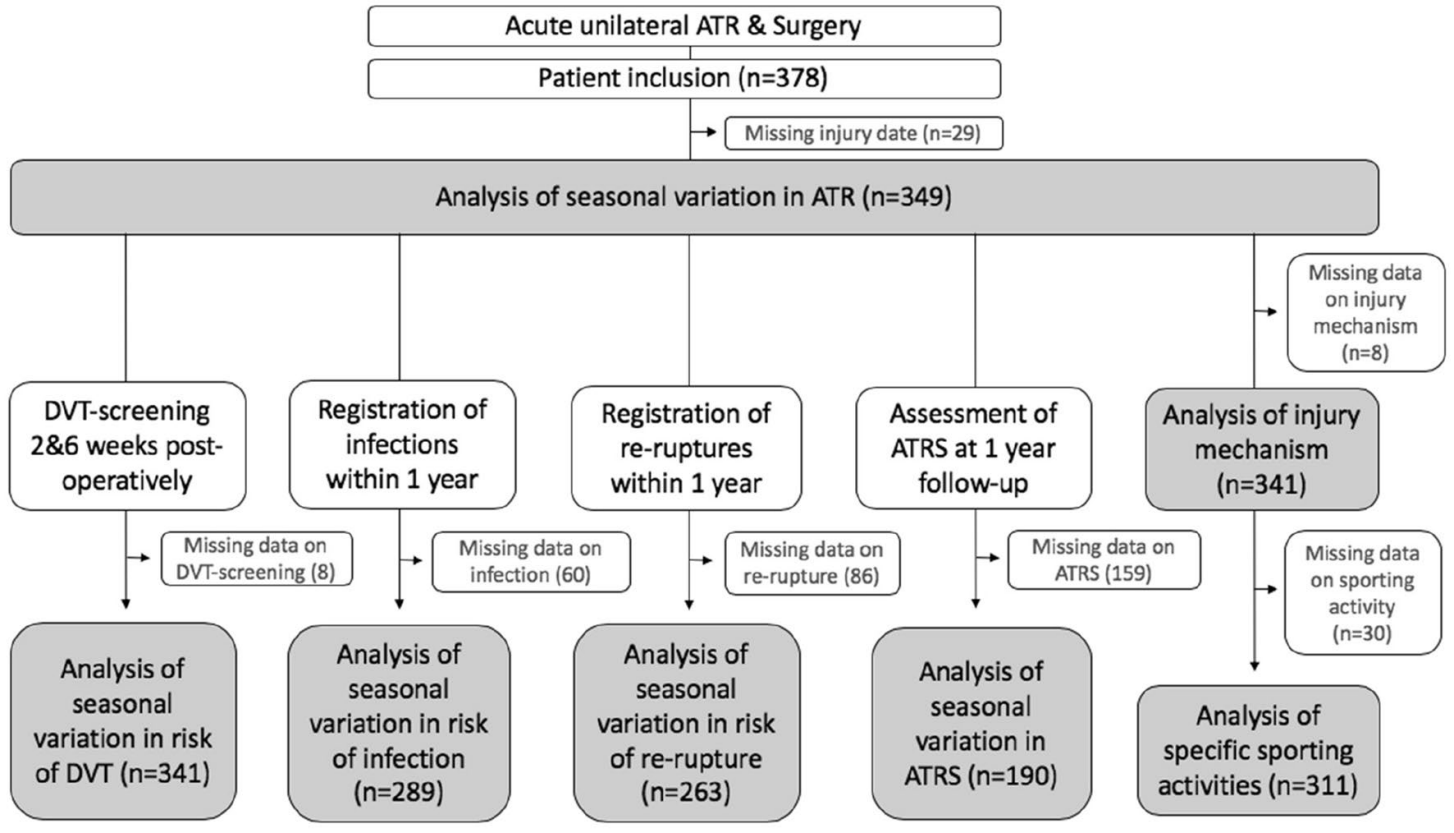
Flow chart. *DVT* deep venous thrombosis, *ATRS* Achilles tendon total rupture score

Selection of study population is described in Fig. 1. This study used data on patients included in two randomized controlled trials (RCTs) investigating different post-operative ATR treatments [1, 6, 7]. The majority of study-patients were operated at Karolinska University Hospital in Solna, Sweden, between 2010 and 2018. Some patients were operated at Danderyd Hospital in Stockholm.

Similar inclusion- and exclusion criteria were used in the available RCTs. Those included were 18–75-year-old patients surgically treated for an acute unilateral ATR within 1 week after injury [1, 6, 7]. Patients with ongoing anticoagulation therapy, known kidney failure, heart failure with pitting oedema, thrombophlebitis, any thromboembolic event during the previous three months, other surgery during the previous month, known malignancy, haemophilia and pregnancy were excluded [1, 6, 7]. Unwillingness to participate, inability to follow instructions or to give informed consent and planned follow-up at another hospital also led to exclusion [1, 6, 7]. Patient characteristics are shown in Table 1.

**Table 1:** Patient characteristics

Variable	Total
Age, mean (SD, range)	39.8 (8.0, 53)
Sex, male, *n* (%)	270 (82.1)
Sex, female, *n* (%)	59 (17.9)
BMI, kg/cm^2^, mean (SD)	26.0 (3.4)
Current smokers, *n* (%)	15 (4.6)

*SD* standard deviation, *BMI* body mass index

### Seasonal variations

Injury dates were grouped into seasons to analyse seasonal differences. Seasons were defined as the meteorological seasons in the northern hemisphere; Spring: March 1st to May 31st, summer: June 1st to August 31st, autumn: September 1st to November 30th, winter: December 1st to February 28th.

### Treatment protocols

Standardized anaesthetic- and surgical techniques were used for all patients [1, 6, 7]. A modified Kessler suture technique was used and no anti-inflammatory or thromboprophylactic drugs were given post-operatively [1, 6, 7]. Patients who developed DVT received low-molecular-weight heparin according to hospital protocol [7].

Four different post-operative treatment protocols were used during 6 weeks after ATR reconstruction and none of the treatments differed significantly between seasons (n.s.). The first group was treated with a plaster cast with the ankle in 30° equinus for 2 weeks and weight bearing was not allowed [1, 6, 7]. The second group was treated with a plaster cast in combination with foot intermittent pneumatic compression (IPC) for 2 weeks and weight-bearing was not allowed [7]. After 2 weeks, a conversion to orthotic treatment with full weight-bearing was done for the first and second treatment groups, and was continued for additional 4 weeks [6, 7]. The third group was treated with an orthosis and calf-IPC for 2 weeks and weight bearing was allowed as tolerated. After 2 weeks, the orthotic treatment continued without calf-IPC for additional 4 weeks [6]. The fourth group was treated with functional weight-bearing mobilization by using an ankle-mobile orthosis (VACOPed) [1]. VACOPed-treatment was continued during the whole 6-week post-operative period.

### Assessment of DVT

All patients were screened on the injured side for DVT at 2 and 6 weeks post-operatively [1, 6, 7]. Compression sonography with colour Doppler was used (Philips CX 50 ultrasound machine; Philips Medical Systems, Andover, Massachusetts, USA) and the screening was done by ultrasonographers blinded to treatment [1, 6, 7]. The diagnostic criteria for DVT has been described earlier [16]. The standard procedure included evaluation of all deep proximal and distal veins [1, 6, 7]. It has been demonstrated that it is difficult for a clinician to differentiate between pain from a rupture/operation, and pain caused by a DVT. Therefore, the symptomatic or asymptomatic nature of the DVTs was not recorded in the trials.

### Assessment of injury mechanism, re-ruptures, infections and treatment factors

The injury mechanism was registered by reviewing medical records. In the majority of medical records the physician had specified the specific sporting activity that caused the ATR. The re-ruptures were registered in the database as they occurred. The wound was inspected at 2 and 6 weeks post-operatively, and a wound swab was taken in case of clinical suspicion of an infection. Infections reported in this study are the culture positive wound swabs. Culture confirmed infections were treated with adequate antibiotics. Treatment factors, including any per-operative complication, operation time, time to operation, the rate of closed paratenon, closed fascia cruris and surgeon categorized as compliant to the predefined operative protocol, were collected by a review of medical records. Any per-operative complication included extension of the incision, difficulties to adapt the tendon ends and consultation of a more senior colleague.

### Patient reported outcome measures

Patient-reported outcome at 12 months post-operatively was evaluated with Achilles tendon total rupture score (ATRS, Swedish, version 6) which is a validated, self-administered questionnaire [19]. ATRS includes 10 questions and 100 is the maximal total score [19]. ATRS > 80 is in this study considered as an overall good patient-reported outcome, and has been used before [26], but this cut-off value is chosen arbitrarily.

### Statistical analysis

All statistical analyses were conducted by SPSS version 25 (IBM Corp.). The sample size for the cohort was calculated on a difference in the rate of ATR of 15% between groups. It was determined that a sample size of 242 in total would be necessary to detect the difference in ATR-rate with 80% power when alpha was set equal to 5%. Three-hundred and forty-nine patients in total were included to account for loss to follow-up and subgroup analyses. All continuous variables were checked for skewness and 95% confidence intervals were derived. Patient characteristics were summarized with standard descriptive statistics such as mean, standard deviation, frequency and percentage. Group differences were analysed with One-way ANOVA for continuous variables and with Pearson´s chi-squared test or Fisher´s exact test for categorical variables. Significance level was set to *p* < 0.050.

## Results

### Seasonal variation in the rate of Achilles tendon ruptures

**Fig. 2:**
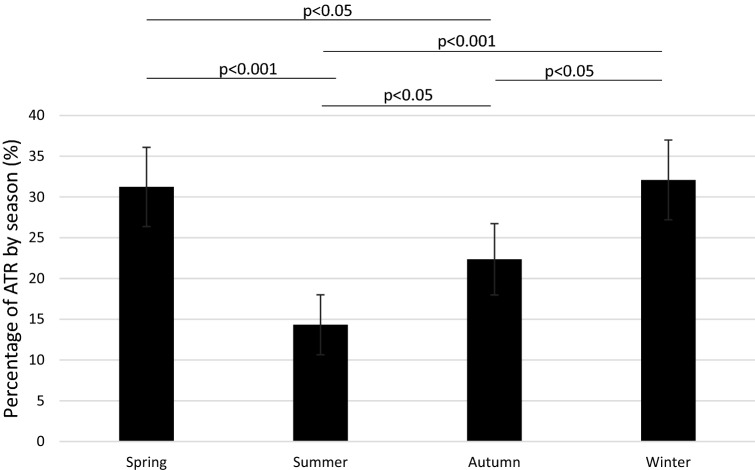
The percentage of patients with an Achilles tendon rupture by season. Chi-squared test, exact significance (2-sided). The difference between spring and winter was not statistically significant (n.s.). 95% Confidence intervals included. *ATR* Achilles tendon rupture

Distribution of patients with an ATR by seasons is shown in Fig. 2. Winter and spring exhibited the highest rate of ATR, while summer exhibited the lowest rate of ATR (*p* < 0.05). The difference in rates of ATR between winter and spring was not statistically significant (n.s.).

### Injury mechanism of ATR

The majority of the ATR-cases (91.5%), were sports-related. The most common sporting activities associated with ATR were badminton (23%), soccer (22%) and floorball (11%). The different sports and the gender distribution among the sports are presented in Table 2.

**Table 2:** Sporting activities causing the most of Achilles tendon ruptures and proportion of females

Sporting activity	Sample size	Percentage	Females, n (%)
Badminton	73	24	9/70 (13)
Soccer	69	22	5/63 (8)
Floorball	33	11	2/31 (6)
Gym^a^	18	6	11/17 (65)
Dance^a^	4	1	2/3 (67)
Volleyball^a^	5	2	3/5 (60)
Other	109	35	21/103 (20)
Total	311	100	53/292 (18)

^a^Sporting activities with female predominance

**Fig. 3:**
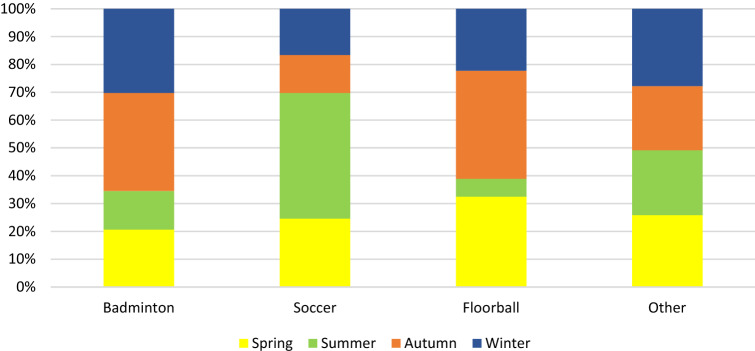
Most common sporting activities associated with Achilles tendon rupture and the proportions by season

Seasonal variation within the sporting activities most associated with ATR are shown in Fig. 3. The proportion of soccer-related ATR was significantly higher during summer compared to autumn, winter or spring (*p* < 0.05). The seasonal differences in the proportions of badminton- and floorball related ATR were not statistically significant (badminton n.s., floorball n.s.).

### Seasonal variation in the risk of adverse events

**Fig. 4:**
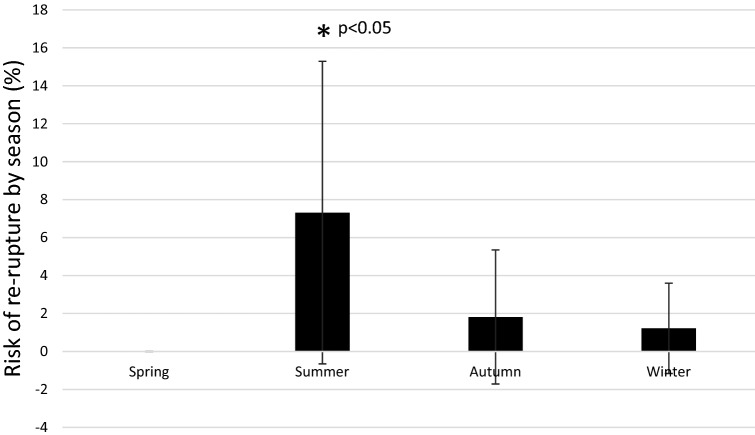
Risk of re-rupture by season. *p* value from chi-squared test. Exact significance (2-sided). The difference between spring, autumn and winter compared to summer was statistically significant (*p* < 0.05). Differences between other seasons were not significant

Patients that sustained an ATR during summer exhibited a significantly higher risk of re-rupture compared to other seasons (*p* < 0.05) (Fig. 4). The total risk of re-rupture was 1.9% (5/263, 95% CI: 0.3–3.6).

In total, 46% of the ATR-patients had a DVT on screening at 2 or 6 weeks post-operatively. The risk of DVT was highest during spring (51%), but differences between seasons were not significant (n.s.) (Table 3). There were no significant seasonal differences in the risk of infection (n.s.) (Table 4).

**Table 3:** Seasonal risk of deep venous thrombosis

Season	Sample size (ATR)	DVT, *n* (%)	95% CI	*p* value
Spring	108	55 (50.9)	41.5–60.3	n.s
Summer	49	23 (46.9)	32.9–60.9	
Autumn	75	33 (44.0)	32.8–55.2	
Winter	109	46 (42.2)	32.9–51.5	
Total	341	157 (46.0)	40.7–51.3	

Chi-squared test, exact significance (2-sided)

**Table 4:** Seasonal risk of infection

Season	Sample size (ATR)	Infection, *n* (%)	95% CI	*p* value
Spring	95	3 (3.2)	− 0.4 to 6.7	n.s
Summer	44	1 (2.3)	− 2.1 to 6.7	
Autumn	62	4 (6.5)	0.3–12.6	
Winter	88	4 (4.5)	0.2–8.9	
Total	289	12 (4.2)	1.9–6.5	

Chi-squared test, exact significance (2-sided)

### Correlations of re-rupture with treatment factors

**Fig. 5:**
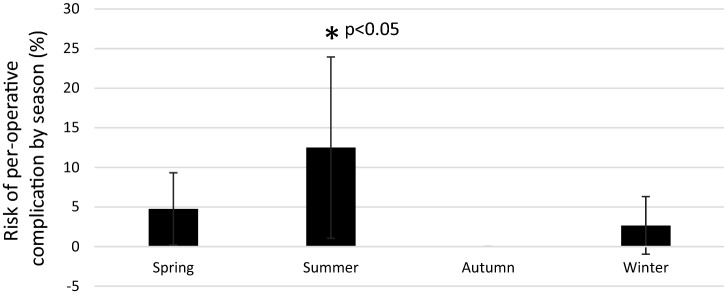
Risk of any documented per-operative complication by season. *P* value from chi-squared test

Any per-operative complication documented in the medical records was more common during summer compared to other seasons (*p* < 0.05) (Fig. 5). Operation time was shorter during winter compared to autumn and spring (*p* < 0.05). Operation time did not significantly differ between summer and other seasons. Time to operation did not differ between seasons (n.s.). The rate of closed paratenon (n.s.), closed fascia cruris (n.s.), using 2 PDS sutures (n.s.) or surgeon categorized as compliant to the standardized surgical protocol (n.s.) did not differ between seasons.

### Seasonal variation in ATRS 12 months post-operatively

**Fig. 6:**
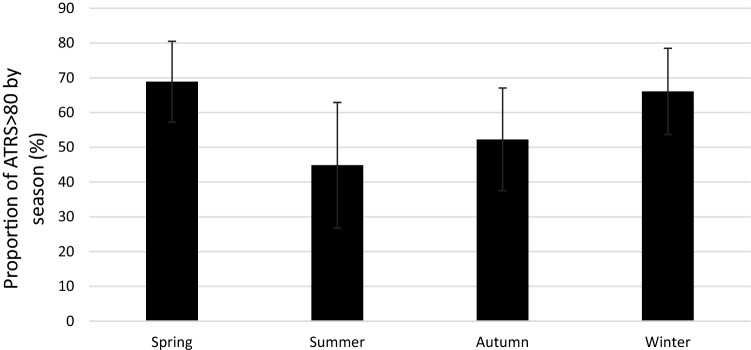
Proportion of overall good patient-reported outcome (ATRS > 80) by season. 95% confidence intervals included. The mean ATRS was 79.6 (SD 18.0)

In total, 61% of patients reported an ATRS > 80, which was considered as an overall good outcome [26]. Patients sustaining an ATR during spring reported the highest rate of an overall good outcome (69%), while patients sustaining an ATR during summer reported the lowest rate (45%). The difference was however not significant (n.s.) (Fig. 6).

## Discussion

This study demonstrated that the rate of Achilles tendon rupture (ATR) related to sports was significantly highest during winter and spring, and lowest during summer in adults in Stockholm, Sweden. Moreover, the risk of sustaining a re-rupture or per-operative complication was higher for patients who sustained their ATR during summer than during other seasons. The incidence of other complications such as deep venous thrombosis (DVT) and infection as well as the proportion of patients with a good patient-reported outcome did not significantly differ between seasons.

The main finding of this study showing that sports-related ATR in the adult population in Stockholm, Sweden, is most common during winter and spring is in accordance with previous evidence from Vancouver, Canada and New York, USA [3, 24]. These studies were also based on surgically treated ATR-patients [3, 24]. Moreover, observation that the seasonal differences only are significant for sports-related activities is also in accordance with earlier evidence [24]. The finding that the non-sports-related ATR only accounted for less than 9% of all ATR injuries also illustrates that the calculation of seasonal differences among non-sports-related injuries would require a considerably larger cohort.

The finding that the rate of ATR injuries was highest during winter and spring is however, not in accordance with a large Danish, general population based study showing highest ATR-incidence during autumn [9]. Discrepancies between the studies may relate to variations in sporting activities between the countries or differences between the cohorts. The cohort from this study is also more comparable with that from Vancouver and New York with respect to that all patients were treated with surgical repair [3, 24].

The result showing that most ATR injuries occurred during winter should be related to the observations that badminton, soccer and floorball were the three most common sporting activities while sustaining an ATR. All these are popular indoor sports during winter in Sweden. This conclusion was further supported by subgroup analyses demonstrating that ATR injuries during winter were, in over 80%, associated with indoor sports: badminton (27%), soccer (17%), gym (10%), floorball (9%), squash (8%), handball (7%) and tennis (6%). The observation, that the rate of ATR injuries during spring was almost as high as the corresponding rate during winter, suggests that the difference in sports-related injuries between these seasons was not as large as expected. The hypothesis was partly verified by demonstrating that ATR injuries during spring were in over 80%, associated with soccer (25%), badminton (19%), floorball (13%), tennis (8%), squash (7%), obstacle course racing (6%) and gym (4%).

Other factors underlying the higher rate of ATR, observed during winter and spring, may be cold weather and exposure to new sports. It has been suggested that a cold Achilles tendon prior to exercise, and sudden increases in load, may predispose for ATR [11]. Moreover, there is a pattern where people with sedentary lifestyle start doing new sports during winter and spring. Based on these suggestions, prevention of ATR in high risk sports may include warm-up injury prevention programmes, similar to what has been done to prevent injuries in e.g. soccer [27]. Such prevention programmes should start 3 months before engaging fully in a new sport and include strength exercises of the Achilles tendon [2, 18].

The finding of badminton, soccer and floorball comprising more than half of the ATR injuries in this cohort would seem to suggest that preventive approaches should be primarily directed towards these specific activities. The observation of the highest proportion of soccer-related ATR during summer, indicates a benefit from preventive approaches, especially during summer, or even during spring. One hypothesis for the high rate of soccer-related ATR during summer may be intermittent soccer players who return to this sport during summer months, as ATR may be more common in intermittently active people [8]. Further studies should test these targeted preventive programmes, especially in people intermittently active in one of these high-risk sports.

Contrary to expected, the lowest rate of infections was seen during summer, but the differences were not significant. Earlier studies have indicated that the incidence of venous thromboembolism, if the underlying cause is not specified, may be higher during winter [4]. In this study, however, the risk of DVT did not demonstrate any significant seasonal differences, which indicate that seasonal factors do not affect the risk of DVT during leg immobilization after ATR surgical repair.

The second main finding of this study was that risk of re-rupture (within 1 year after surgery) was higher for patients sustaining their ATR during summer than during other seasons. The finding that patients sustaining an ATR during summer exhibited a trend towards lower scores in the patient-reported outcome would seem to strengthen the notion above. The higher risk of re-rupture during summer may be reflective of several factors. The finding that the incidence of infections and DVTs did not vary over seasons would seem to indicate, that the risk of re-rupture is not correlated with the risk of infection/DVT.

The procedure included in ATR-care comprises of waiting time to surgery, time during surgery, surgical procedures and post-operative care. Additional analyses demonstrated that any documented per-operative complication reported in the medical records was significantly more common during summer than during other seasons, which may explain the higher risk of re-rupture. Therefore, it may be that ATR-care holds a lower quality during summer months. Another explanation could be, that the risk of per-operative complication, and re-rupture may be increased by a more complicated rupture, due to different injury mechanisms during summer. In this study, soccer was the most prominent injury mechanism during summer. However, none of the re-ruptures in this study (3/5 were sports-related) occurred in patients with soccer related injuries. Another factor influencing the outcome could be, that patients are more likely to discontinue their orthotic treatment during summer. Therefore, further studies with larger cohorts will be necessary to draw final conclusions on the aetiology of seasonal risk factors for re-rupture.

This study had several advantages that enabled a valuable analysis. All patients were screened for DVT post-operatively, which gave a unique possibility to also include asymptomatic DVT-cases. The number of ATR-cases with an injury date available was relatively high, which increased the power to show seasonal differences. Data were collected during several years, which may have evened out potential monthly and seasonal variations in the efficacy of patient recruitment. Post-operative treatment protocols used did not significantly differ between seasons. Therefore, the seasonal risk of DVT, re-rupture or poor patient-reported outcome was not suspected to be influenced by different post-operative treatment protocols. The age of the included patients did not significantly differ between seasons.

This study had some limitations that must be considered when interpreting the results. In this study, only surgically treated patients were included. The predominant number of ATR patients was, however, surgically treated since there were ongoing randomized clinical trials including surgical repair and no standard non-operative treatment was established. The exact number of ATR-cases per season may partly depend on the efficacy of patient recruitment, which might have varied between months and seasons. Especially during summer, the recruitment was less effective because of the lower resources. The patient reported outcome measure may have been affected by non-standardized rehabilitation between 6 weeks and 1 year. All the included cohorts did not use ATRS as primary outcome, which resulted is a lower rate of patients with ATRS. The study population was selected from hospitals in Stockholm and therefore the generalizability of the results may be restricted. The numbers of infections and re-ruptures were also low, which must be considered when interpreting those results.

## Conclusion

Winter and spring are high risk seasons for sports-related ATR and the risk sports are badminton, soccer and floorball. Risk of re-rupture is highest after surgical ATR repair during summer and the reason should be further investigated, in order to provide equal care during all seasons.

## Abbreviations

ATR: Achilles tendon rupture
DVT: Deep venous thrombosis
ATRS: Achilles tendon Total Rupture Score
